# Prefrontal and Striatal Activity Related to Values of Objects and Locations

**DOI:** 10.3389/fnins.2012.00108

**Published:** 2012-07-17

**Authors:** Soyoun Kim, Xinying Cai, Jaewon Hwang, Daeyeol Lee

**Affiliations:** ^1^Department of Neurobiology, Yale University School of MedicineNew Haven, CT, USA; ^2^Department of Anatomy and Neurobiology, Washington University School of MedicineSt. Louis, MO, USA; ^3^Department of Brain and Cognitive Sciences, University of RochesterRochester, NY, USA; ^4^Zanvyl Krieger Mind/Brain Institute, John Hopkins UniversityBaltimore, MD, USA

**Keywords:** intertemporal choice, prefrontal cortex, reward, temporal discounting, utility

## Abstract

The value of an object acquired by a particular action often determines the motivation to produce that action. Previous studies found neural signals related to the values of different objects or goods in the orbitofrontal cortex, while the values of outcomes expected from different actions are broadly represented in multiple brain areas implicated in movement planning. However, how the brain combines the values associated with various objects and the information about their locations is not known. In this study, we tested whether the neurons in the dorsolateral prefrontal cortex (DLPFC) and striatum in rhesus monkeys might contribute to translating the value signals between multiple frames of reference. Monkeys were trained to perform an oculomotor intertemporal choice in which the color of a saccade target and the number of its surrounding dots signaled the magnitude of reward and its delay, respectively. In both DLPFC and striatum, temporally discounted values (DVs) associated with specific target colors and locations were encoded by partially overlapping populations of neurons. In the DLPFC, the information about reward delays and DVs of rewards available from specific target locations emerged earlier than the corresponding signals for target colors. Similar results were reproduced by a simple network model built to compute DVs of rewards in different locations. Therefore, DLPFC might play an important role in estimating the values of different actions by combining the previously learned values of objects and their present locations.

## Introduction

During decision making, outcomes expected from different actions are evaluated along multiple dimensions, including various properties of the object targeted by each action and the cost of acquiring it. The relative importance of each dimension is likely to vary according to the nature of the task at hand. For example, when the same products are available at the same price in multiple stores, our choice would be largely governed by the differences in their locations. Indeed, neural signals related to the subjective values of actions directed to different target locations are widespread in the brain, ranging from the posterior parietal cortex (Platt and Glimcher, 1999; Dorris and Glimcher, 2004; Sugrue et al., 2004; Seo et al., 2009) and prefrontal cortex (Barraclough et al., 2004; Kim et al., 2008) to the striatum (Samejima et al., 2005; Lau and Glimcher, 2008; Kim et al., 2009b; Cai et al., 2011). In some cases, however, the decisions might be made between different objects or goods before their locations are taken into consideration, as when a shopper tries to determine which item to purchase before considering the location of its store (Padoa-Schioppa, 2011). Previous neuroimaging studies have also shown that the human ventromedial prefrontal cortex (VMPFC) is activated according to the value of the chosen option, regardless of whether the actions necessary to acquire an object are known in advance or not (Boorman et al., 2009; Wunderlich et al., 2009, 2010). Similarly, activity in the primate orbitofrontal cortex largely reflects the value of an object regardless of its location or required actions (Tremblay and Schultz, 1999; Wallis and Miller, 2003; Padoa-Schioppa and Assad, 2006). However, how these abstract value signals contribute to choosing appropriate actions is largely unknown.

Several lines of evidence suggest that this transformation might be mediated by the dorsolateral prefrontal cortex (DLPFC). First, neurons in the DLPFC encode the value of expected reward (Roesch and Olson, 2003; Kennerley and Wallis, 2009), often for specific actions (Watanabe, 1996; Leon and Shadlen, 1999; Barraclough et al., 2004; Kim et al., 2008). Second, the orbitofrontal cortex and DLPFC are connected reciprocally (Barbas and Pandya, 1989; Carmichael and Price, 1996), and this might provide the anatomical substrates for the conversion between the value signals associated with specific objects and actions. Previously, we have shown that during intertemporal choice, neurons in the DLPFC and striatum modulate their activity according to the temporally discounted values (DVs) of rewards expected from different actions (Kim et al., 2008; Cai et al., 2011). However, whether these two structures also play any role in extracting such action-based value signals from the information about the values of different objects and their locations has not been investigated. In the present study, we found that a small but significant proportion of neurons in both of these areas encoded the signals related to the values of rewards expected from distinct visual objects. These results suggest that the DLPFC and striatum might be a part of the network involved in utilizing the values encoded in spatial and non-spatial frames of reference to guide the animal’s actions.

## Materials and Methods

### Animal preparations

Three rhesus monkeys (D, H, and J) were trained to perform an oculomotor intertemporal choice task described previously (Hwang et al., 2009). All the methods used to collect the behavioral and neural data analyzed in this study have been previously described (Kim et al., 2008; Cai et al., 2011). Neurons in the DLPFC were recorded from all three animals (Kim et al., 2008), whereas the neurons in the striatum were recorded in two animals (monkeys J and H; Cai et al., 2011). In all experiments, the animal’s eye position was monitored with a video-based eye tracking system with a 225 Hz sampling rate (ET-49, Thomas Recording, Giessen, Germany). Single-neuron activity was recorded from the DLPFC, caudate nucleus or ventral striatum, using a multielectrode recording system (Thomas Recording) and multi-channel acquisition processor (Plexon, Inc., Dallas, TX, USA). All the procedures used in this study were approved by the University of Rochester Committee on Animal Research and the Institutional Animal Care and Use Committee at Yale University, and conformed to the Public Health Service Policy on Humane Care and Use of Laboratory Animals and the Guide for the Care and Use of Laboratory Animals.

### Behavioral task

**Figure 1:**
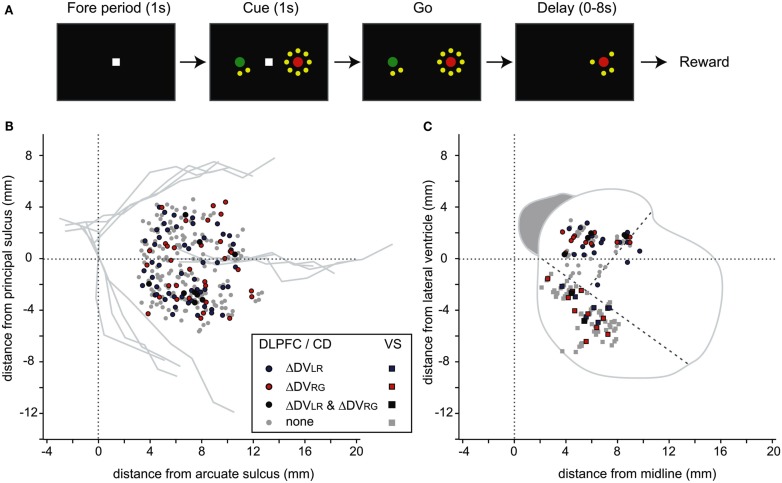
**Behavioral task and recording locations**. **(A)** Temporal sequence of intertemporal choice task. **(B)** Recording locations for the neurons in the DLPFC that modulated their activity according to the difference in the temporally discounted values related to the targets in different positions (ΔDV_LR_) or in different colors (ΔDV_RG_). Horizontal (anterior-posterior) distance is measured relative to the arcuate sulcus, whereas the vertical (dorso-ventral) distance is relative to the principal sulcus. **(C)** Recording locations for the neurons recorded in the CD and VS. Oblique dotted lines indicate approximately the borders between the CD, VS, and putamen.

A trial began when the animal fixated a small white square at the center of a computer screen. Following a 1-s fore-period, two peripheral targets were presented along the horizontal meridian (Figure 1A). One target was green and delivered a small reward (0.2 or 0.26 ml of apple juice) when chosen by the animal, while the other target was red and delivered a large reward (0.4 ml). The animal was required to indicate its choice by shifting its gaze toward its chosen target when the central fixation target was extinguished after a 1-s cue period. The delay between the animal’s choice and reward delivery was indicated separately for each target by the number of yellow disks (1 s/disk). For some neurons recorded in the DLPFC (*n* = 185), cyan disks were also used to indicate the reward delays in some trials (4 s/disk; for more details, see Kim et al., 2008). Once the animal shifted its gaze toward one of the peripheral targets, disks around the chosen target were extinguished individually (1 and 4 s/disk for yellow and cyan disks, respectively), and the reward was delivered when the last disk was removed. Following a trial when the animal chose the small reward target, the intertrial interval was increased by the difference in the reward delays for the small and large reward so that the onset of the next trial was not affected by the animal’s choice. All neurons recorded in the striatum and a subset of DLPFC neurons were further tested during a control task in which the color of the central fixation target (red or green) indicated the target the animal was required to choose. During this control task, the animal received a small reward (0.13 ml) without any delay for its correct performance.

### Analysis of behavioral data

For each of the two targets in a given trial, its temporally discounted value (DV) was computed using a hyperbolic discount function as follows,

$$\text{D}{\text{V}}_{x}=\frac{A_{x}}{\left(1+kD_{x}\right)},$$

where *A_x_* and *D_x_* denote the magnitude and delay of reward available from target *x*. For comparison, we have also tested an exponential discount function, which is given by the following.

$$\text{D}{\text{V}}_{\text{x}}=A_{x}\exp\left(-kD_{x}\right).$$

The probability of choosing the target (*C* = L or R, for leftward and rightward choices) was then given by the logistic transformation of the difference in the DVs of the two targets, namely,

$$P\left(C\text{ = L}\right)=\sigma \left[\beta \Delta D{\text{V}}_{\text{LR}}\right],\text{and }P\left(C\text{ = R}\right)\text{1}-P\left(C\text{ = L}\right),$$

where ΔDV_LR_ ≡ DV_Left_ − DV_Right_ denotes the difference in the DVs for the left (DV_Left_) and right (DV_Right_) targets, σ[*z*] = {1 + exp(−*z*)}^−1^ corresponds to the logistic transformation, and β is the inverse temperature parameter. The model parameters (*k* and β) were estimated using the maximum likelihood procedure as in the previous studies (Kim et al., 2008; Hwang et al., 2009). Model performance for hyperbolic and exponential discount functions was evaluated using the likelihood of each model, since the number of free parameters was the same for the two models.

### Analysis of neural data

How activity of the neurons described in the present study might be related to the DVs for the rewards available from specific target locations was reported previously (Kim et al., 2008; Cai et al., 2011). As in those studies, we analyzed the spike rates of each neuron during the 1-s cue period using a series of regression analyses. In the present study, we tested whether the activity of each neuron was modulated by the difference in the DVs for left and right targets or by their difference for red (large reward) and green (small reward) targets, using the following model,

(model 1) $$\begin{array}{rcll} S & =a_{\text{0}}+a_{\text{1}}\Delta D{\text{V}}_{\text{LR}}+a_{\text{2}}\Delta DV{\text{L}}_{\text{RG}}+a_{\text{3}}\Delta D{\text{V}}_{\text{CU}} & & \\ & \;+a_{\text{4}}\text{D}{\text{V}}_{\text{sum}}+a_{\text{5}}C+a_{\text{6}}C^{*}, & & \end{array}$$

where *S* indicates the spike count during the cue period, ΔDV_RG_ ≡ DV_Red_ − DV_Green_ the difference in the DVs for the red (large reward; DV_Red_) and green (small reward; DV_Green_) targets, ΔDV_CU_ ≡ DV_C_ − DV_U_ the difference in the DVs for the chosen (DV_C_) and unchosen (DV_U_) targets, DV_sum_ the sum of the DVs for the two targets, *C* and C* are dummy variables indicating whether the animal chose the leftward (*C* = 0) or rightward (*C* = 1) target and whether the animal chose the green (C* = 0) or red (C* = 1) target, respectively, and *a*_0_ ~ *a*_6_ the regression coefficients. By including DV_sum_ as a separate regressor, this model distinguishes activity changes associated with the differences in the DVs along two different dimensions. Compared to the regression models used in our previous studies (Kim et al., 2008; Cai et al., 2011), the above model includes the term for the difference in the DVs for the red and green targets (ΔDV_RG_) and the dummy variable corresponding to the color of the target chosen by the animal (C*). In this model, neural activity related to the values and choices in the spatial frame of reference can be estimated separately from those related to different target colors, because the positions of green and red targets were randomized across trials. For the analysis of neural data, we have used the hyperbolic discount function to estimate the DVs in each trial, since it tended to account for the animal’s choice behavior better than the exponential discount function (Kim et al., 2008; Hwang et al., 2009; see Results). None of the results reported below changed significantly, however, when we used the DVs estimated using the exponential discount function for the sessions in which it accounted for the animal’s behavior better than the hyperbolic discount function.

To compare the time course of neural activity related to the DVs associated with target colors and actions, we applied this regression model to the spike rates estimated during a 200-ms window sliding at 25 ms steps, and computed for each variable the coefficient of partial determination (CPD; Kim et al., 2008). CPD was used to quantify how strongly the activity of a given neuron was modulated by a particular variable. The CPD for *X*_i_ is defined as the following.

$$\text{CPD(}X_{\text{i}}\text{)}=\frac{\left\{\text{SSE}\left(X_{-\text{i}}\right)-\text{SSE}\left(X_{-\text{i}},X_{\text{i}}\right)\right\}}{\text{SSE}\left(X_{-\text{i}}\right)},$$

where SSE (*X*) refers to the sum of squared errors in a regression model that includes a set of regressors *X*, and *X*_-i_ a set of all the regressors except *X*_i_. For the neurons in which the effect of a given variable was significant for the entire cue period, its latency was defined as the first time when the CPD for that variable exceeded for three consecutive windows (75 ms) four times its standard deviation above its baseline estimated during 1 s before cue onset.

If the activity of a given neuron is reliably modulated by the difference in the DVs for the red and green target, as evidenced by the corresponding regression coefficient (*a*_2_) that is significantly different from 0, this might reflect exclusively the effect of the DV of just one target. Therefore, to further test whether and how the activity of each neuron was modulated by the DVs of both targets independent of their locations, we applied the following model.

(model 2) $$\begin{array}{rcll} S & =a_{\text{0}}+a_{\text{1}}\text{D}{\text{V}}_{\text{red}}+a_{\text{2}}\text{D}{\text{V}}_{\text{green}} & & \\ & \;+a_{\text{3}}\Delta D{\text{V}}_{\text{LR}}+a_{\text{4}}\Delta D{\text{V}}_{\text{CU}}+a_{\text{5}}C+a_{\text{6}}C^{*}.\; & & \end{array}$$

Previously, we showed that neurons in the DLPFC and striatum encode DVs by combining signals related to reward magnitude and delay (Kim et al., 2008; Cai et al., 2011). Here, to test whether these neurons also encoded the reward delays associated with the red and green targets in addition to the delays associated with left and right targets, we applied the following regression model.

(model 3) $$\begin{array}{rcll} S & =a_{\text{0}}+a_{\text{1}}\Delta D_{\text{LB}}+a_{\text{2}}\Delta D_{\text{RG}}+a_{\text{3}}\Delta D_{\text{CU}} & & \\ & \;+a_{\text{4}}\Delta D_{\text{sum}}+a_{\text{5}}M+a_{\text{6}}C+a_{\text{7}}C^{*}, & & \end{array}$$

where Δ*D*_LR_ ≡ *D*_Left_ − *D*_Right_ denote the difference in the reward delays for the left (*D*_Left_) and right (*D*_Right_) targets, Δ*D*_RG_ ≡ *D*_Red_ − *D*_Green_ the difference in the reward delays for the red (*D*_Red_) and green (*D*_Green_) targets, Δ*D*_CU_ the difference in the reward delays for the chosen (*D*_C_) and unchosen (*D*_U_) targets, *D*_sum_ is the sum of the reward delays for both targets, and *M* the position of the large reward target (0 and 1 for left and right, respectively). We also tested whether the activity seemingly related to DVs simply reflected the features of visual stimuli used to indicate reward magnitude and delays by applying the following model to the neurons tested in the control task.

(model 4) $$\begin{array}{rcll} S & =a_{\text{0}}+a_{1}\Delta D{\text{V}}_{\text{LR}}+a_{2}\Delta D{\text{V}}_{\text{RG}}+a_{3}\Delta D{\text{V}}_{\text{CU}}+a_{4}\Delta D{\text{V}}_{\text{sum}} & & \\ & \;+a_{5}C+a_{6}C^{*}+a_{7}T+a_{8}T\times \Delta \text{D}{\text{V}}_{\text{LR}}+a_{8}T\times \Delta D{\text{V}}_{\text{RG}} & & \\ & \;+a_{9}T\times \Delta D{\text{V}}_{\text{CU}}+a_{10}T\times \Delta D{\text{V}}_{\text{sum}}+a_{11}T\times C & & \\ & \;+a_{12}T\times C^{*}, & & \end{array}$$

where *T* denotes the behavioral task (0 and 1 for the control and intertemporal choice tasks, respectively). For control trials, DVs were in fact constant, so the values of DV in the above model was substituted by the fictitious DVs computed using the values indicated by the same visual stimuli during the intertemporal choice task.

There are two reasons why the differences in the DVs or reward delays were used throughout the above analyses rather than the corresponding values of individual targets themselves. First, the DVs or reward delays of left and right targets were linearly related to the DVs of red and green targets (i.e., DV_Left_ + DV_Right_ = DV_Red_ + DV_Green_), so it is not possible to estimate the neural activity related to the DVs according to colors and positions of individual targets separately. Second, the animal’s choice was ultimately determined by the difference in the DVs, rather than the DVs of individual targets or their sum. Therefore, the neural activity related to the difference in the DVs is behaviorally more relevant than the DVs of individual targets. Regression analyses and related statistical tests were performed using Matlab (Mathworks, Inc., Natick, MA, USA).

### Simulation of network model

Previous modeling studies has suggested that the transformation from the object-based value signals to action-based value signals might be mediated by a pool of neurons that conjunctively code the value of an object and its position (Soltani and Wang, 2006). To test whether this transformation can be accomplished by the types of neurons identified in the DLPFC and striatum, we modified and tested the model proposed by Soltani and Wang (2006). This model consisted of 11 neurons, of which 8 were excitatory and 3 inhibitory, and was designed to compute the reward values for targets in two different locations (e.g., left vs. right) from the values associated with two different target colors (e.g., red vs. green). Two of the excitatory neurons received fixed inputs, and encoded the magnitude of red and green targets, respectively (see Figure 7A). A set of four excitatory neurons received the additional inputs indicating the relative positions of the red and green targets, and they influenced the activity of the two remaining excitatory neurons encoding the values of targets in different locations. The activity *y*_i_ of each model neuron i was given by the following.

$$\tau \frac{dy_{\text{i}}}{\text{d}t}=-y_{\text{i}}+\sigma \left[\Sigma_{\text{j}}w_{\text{ij}}y_{\text{j}}\text{ + }b\right].$$

where σ[*z*] = {1 + exp(−*z*)}^−1^, *w*_ij_ denotes the connection strength from neuron j to neuron i (*w* = 2 for excitatory connections, and −2 or −4 for inhibitory connections), and *b* a bias input (*b* = 2), and the time constant τ = 100 ms.

## Results

### Activity related to values of objects and locations

We analyzed the activity of 349 DLPFC neurons recorded during the intertemporal choice task (Figure 1B). In addition, a smaller number of neurons were recorded in the caudate nucleus (93 neurons) and ventral striatum (90 neurons). The animals tended to choose the smaller reward more often when its delay was short, and also as the delay for the large reward increased. We have shown that such behaviors were relatively well accounted for by a temporal discounting model, in which the value of the reward from each target is determined by the product of the reward magnitude and a temporal discount function (Kim et al., 2008; Hwang et al., 2009). In addition, the choice behaviors of all three animals tested in this study were better accounted for by hyperbolic discount functions than exponential ones. The proportion of sessions in which the hyperbolic discount function provided a better fit than the exponential function was 72.1% (49/68), 64.2% (86/134), and 94.4% (220/233) in monkeys D, H, and J, respectively.

To separate the neural signals related to the values computed for different target colors and locations, we applied a regression model (model 1) that includes the difference in the DVs for left and right targets (ΔDV_LR_) and the difference in DV for red and green targets (ΔDV_RG_) as well as the sum of the DVs for the two targets (DV_sum_). This model also included the difference in DV for chosen and unchosen rewards (ΔDV_CU_), in addition to two dummy variables indicating whether the animal chose the left or right target (*C*) and whether it chose the red or green target (C*). Therefore, activity related to DVs were separated from the activity related to the binary choice of the animal.

**Figure 2:**
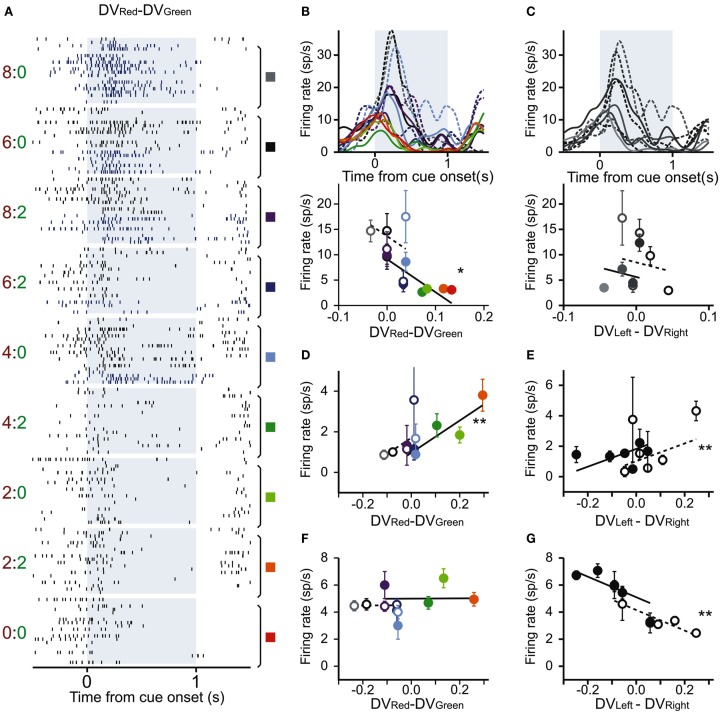
**Three example DLPFC neurons with activity related to temporally discounted values (DVs)**. **(A–C)** A DLPFC neuron that modulated its activity according to the difference in the DVs for red and green targets. **(A)** Raster plots sorted according to the difference in the DVs between red (large reward) target and green (small reward) target (DV_Red_ − DV_Green_). A pair of number to the left indicate reward delays for the red and green targets. Blue and black rasters show the action potentials during the trials in which the animal chose the small and large reward targets, respectively. Colored rectangles and vertical line segments indicate the trials grouped for the results shown in **(B)**. **(B)** Spike density functions (SDF; top) and average firing rates during cue period (bottom) sorted by the difference in the DVs for red (large reward) and green (small reward) targets. Dotted (solid) lines and empty (filled) symbols represent SDF and average firing rates during the trials in which the animal chose the small (large) reward. **(C)** SDF and average firing rates of the same neuron shown in **(A)**, estimated according to the difference in the DVs for left and right targets (represented in the grayscale). Dotted (solid) lines and empty (filled) symbols represent SDF and average firing rates during the trials in which the animal chose left (right) targets. **(D–G)** Average firing rates of two other DLPFC neurons. **(D,E)** Another neuron showing significant modulation in their activity related to the difference in the DVs for the large and small reward targets **(D)** and for the left and right targets **(E)**. **(F,G)** A third neuron showing significant activity only for the difference in DVs of left and right targets. **(D,E–G)** are in the same format as in the bottom panel of **(B,C)**. Error bars, SEM. *p < 0.01; **p < 0.001 (*t*-test for the corresponding regression coefficient).

**Figure 3:**
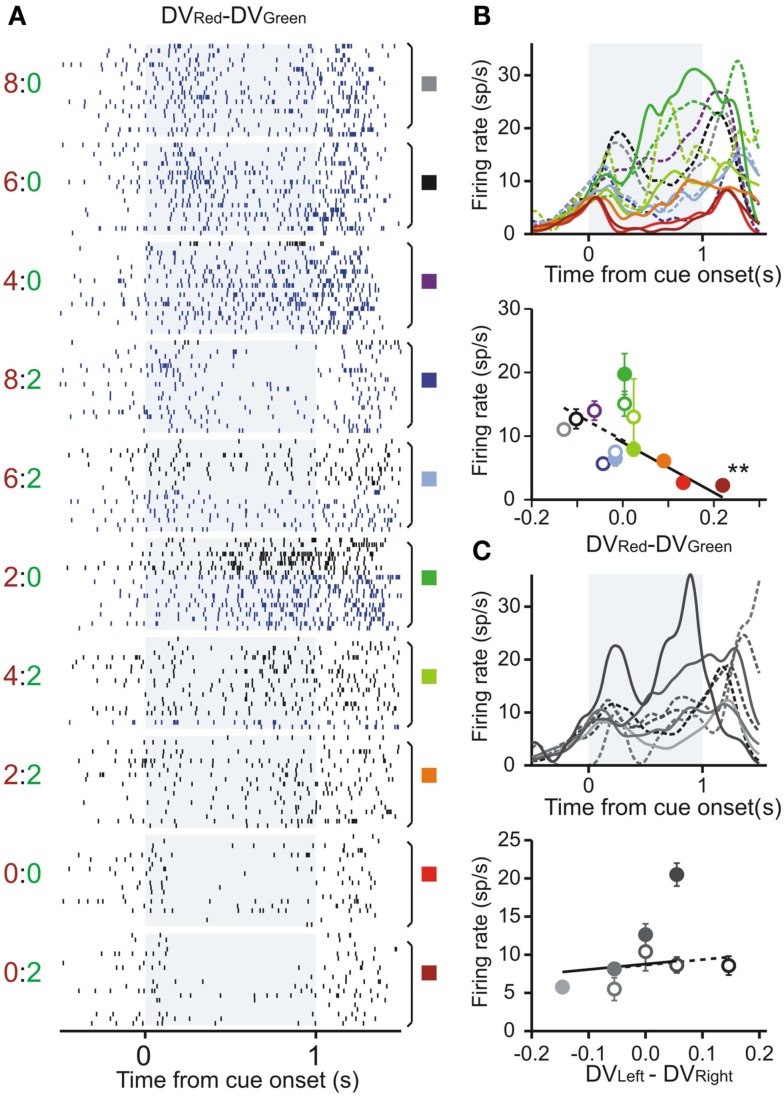
**Example neuron in the ventral striatum encoding the difference in the temporally discounted values for the red and green target**. **(A)** Raster plots sorted according to the difference in the DVs between red and green targets. **(B,C)** Spike density functions and average firing rates during the cue period sorted by the difference in the DVs between red and green targets **(B)** and between left and right targets **(C)**. Same format as in Figures 2A–C.

**Figure 4:**
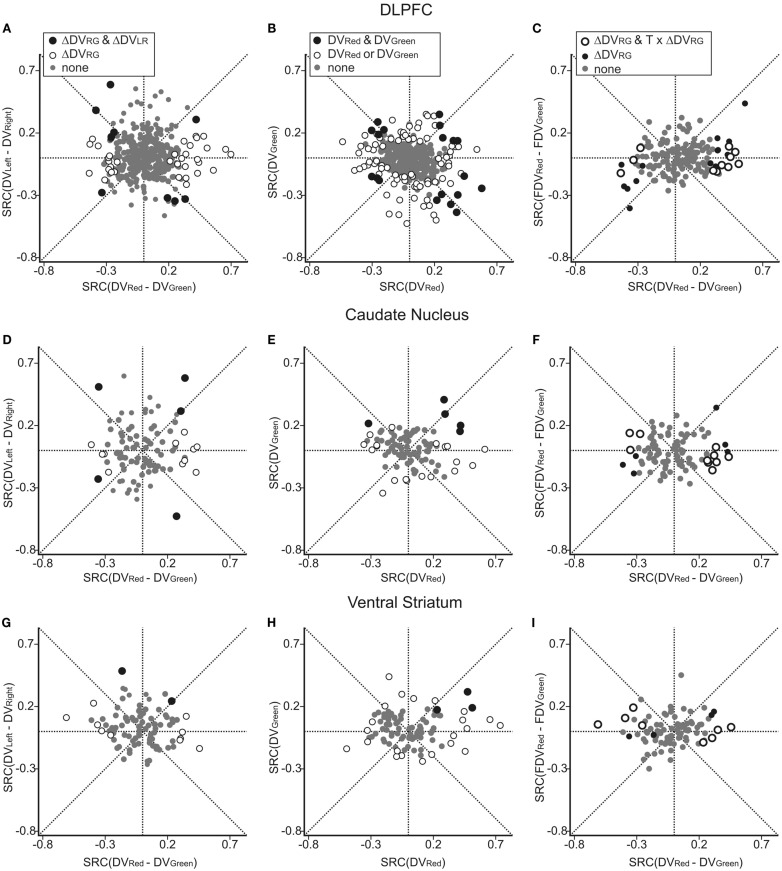
**Neural activity related to temporally discounted values (DVs) in the DLPFC (A–C), CD (D–F), and VS (G–I)**. In **(A,D,G)**, the standardized regression coefficients (SRC) associated with the difference in the DVs for left and right targets (ΔDV_LR_ = DV_Left_ − DV_Right_) are plotted against the SRC associated with the difference in the DVs for red and green targets (ΔDV_RG_ = DV_Red_ − DV_Green_). Empty symbols, neurons showing significant effects of ΔDV_RG_. Black symbols, neurons showing significant effects of both variables. In **(B,E,H)**, SRC for the DVs for red and green targets are plotted against each other. Black and empty symbols, neurons showing significant effect of DVs for both red and green targets and for only one of the two targets, respectively. In **(C,F,I)** SRC for the difference in the DVs for red and green targets during choice trials (ordinate) are plotted against the SRC for the difference in the fictitious DVs for red and green targets during control trials (FDV_Red_ − FDV_Green_). Empty circles, neurons showing significant effect of ΔDV_RG_ and significant interaction between ΔDV_RG_ and the task (model 4); black filled symbols, neurons showing significant effect of ΔDV_RG_ without significant interaction with task.

Using this regression model, we found that neurons significantly modulating their activity according to the difference in the DVs of red and green targets were present in all three regions tested in this study (Table 1). In the DLPFC, 44 neurons (12.6%) significantly changed their activity during the cue period according to the difference in the DVs for red and green targets (Figures 2A–C), whereas 66 (18.9%) neurons significantly changed their activity according to the difference in the DVs for the left and right targets (Figures 2D–G). Similarly, 16 (17.2%) and 12 (13.3%) neurons in the CD and VS also displayed significant modulation in their activity during the cue period related to the difference in the DVs of red and green targets (Figure 3). As reported previously (Cai et al., 2011), the activity related to the DVs of targets in different locations was found significantly more frequently in the CD (24 neurons, 25.8%) than in the VS (nine neurons, 10.0%; χ^2^-test, *p* < 0.01). The number of neurons significantly modulating their activity according to the difference in the DVs was significantly higher than expected by chance (binomial test, *p* < 0.05) in all areas, regardless of whether the values related to left or right target or those related to red and green targets were considered. The number of neurons modulating their activity significantly according to both types of value signals was nine, five, and two for the DLPFC, CD, and VS, respectively (Figure 4, left column). The null hypothesis that these two different types of value signals were combined independently could not be rejected in any of the areas tested in the present study (χ^2^-test, *p* > 0.4). In addition, neurons encoding the difference in the DVs for different target locations were intermixed anatomically along with those encoding the difference in the DVs for different target colors (MANOVA, *p* > 0.15; Figures 1B,C).

**Table 1:** **Number of neurons related to the animal’s choice and temporally discounted values in the DLPFC, caudate nucleus, and ventral striatum**.

	ΣDV	ΔDV_LR_	ΔDV_RG_	ΔDV_CU_	*C*	C*	Total
DLPFC	71 (20.3)	66 (18.9)	44 (12.6)	56 (16.1)	77 (22.1)	56 (16.1)	349
CD	16 (17.2)	24 (25.8)	16 (17.2)	6 (6.5)	24 (25.8)	22 (23.7)	93
VS	24 (26.7)	9 (10.0)	12 (13.3)	12 (13.3)	6 (6.7)	18 (20.0)	90

ΣDV, sum of temporally discounted values; ΔDV_LR_ (ΔDV_RG_, ΔDV_CU_) difference in the temporally discounted values for left and right targets (red and green targets, chosen and unchosen targets, respectively); *C* (C*), animal’s choice between left and right targets (red and green targets).

*The numbers in the parentheses are the percentages*.

Previously, we have also shown that the signals related to the DVs for the targets in different locations were combined heterogeneously across different neurons in the DLPFC and striatum (Kim et al., 2008; Cai et al., 2011). In the present study, we further examined how the signals related to the values of red and green targets were combined across different neurons (Figure 4, middle column). This was accomplished using a regression model that includes the DVs for red and green targets separately (model 2). The results showed that in the DLPFC, 66 (18.9%) and 52 (14.9%) neurons significantly modulated their activity according to the DVs for green and red targets, respectively. A total of 19 neurons changed their activity significantly according to the DVs of rewards for both targets, which was higher than expected if these two signals were combined independently (χ^2^-test, *p* < 0.0005). Among these 19 neurons, activity was affected oppositely by the values of the two targets for 11 neurons. The results for the CD were qualitatively similar. The number of neurons showing significant modulations related to the DVs of red and green target were 12 and 17, respectively. In addition, activity of five neurons was significantly related to the DVs for both red and green targets, and this was significantly more than expected from combining these two signals independently (χ^2^-test, *p* < 0.05). The signs of regression coefficients for the red and green targets were opposite only in one CD neuron. For the VS, the number of neurons showing significant modulation for both red and green targets (*n* = 3) was not significantly higher than expected when the signals related to the value of individual targets are combined independently (χ^2^-test, *p* = 0.50), and they all increased their activity with the DVs of both targets (Figure 4H).

In all three areas tested in the present study, many neurons significantly modulated their activity according to the sum of the DVs associated with the two options in each trial. The percentage of such neurons was 20.3, 17.2, and 26.7% in the DLPFC, CD, and VS (Table 1). Similarly, the neural signals related to the color of the chosen target were found in all three areas (Table 1). In addition, the percentage of neurons showing significant changes in their activity related to the difference in the DVs for the chosen and unchosen options was 16.1, 6.5, and 13.3% in the DLPFC, CD, and VS, respectively. This was significantly above the chance level (5%) in the DLPFC and VS (binomial test, *p* < 0.05), but not in the CD (Table 1).

In the present study, the magnitudes of large and small reward were fixed, and the DV of the large or small reward was entirely determined by its delay. Therefore, we tested whether neurons in the prefrontal cortex and striatum encoded the difference in the delays for the large and small rewards in addition to the delays for the rewards available in different locations. The results of this regression analysis (model 3) showed that the proportion of neurons encoding the difference in the delays for the large and small reward was 15.5, 10.8, and 11.8% for DLPFC, CD, and VS, respectively. The proportion of neurons encoding the delays for the rewards associated with the left and right targets was 17.5, 20.4, and 7.5% for the same areas. These percentages were significantly higher than the chance level (binomial test, *p* < 0.05), except for the signals related to delays for rewards in different locations in the VS.

### Effects of visual stimuli on neural activity

All the neurons recorded in the striatum and 164 neurons recorded in the DLPFC were also tested in control trials in which the same peripheral visual stimuli were presented as in the intertemporal choice task but the animal was required to shift its gaze to a particular target according to the color of the central fixation target (Kim et al., 2008; Cai et al., 2011). Comparison of the neural activity recorded in these two different types of trials indicated that the activity in the DLPFC and striatum related to the DVs of red and green targets did not merely reflect the physical properties of visual stimuli used to indicate reward delays during the intertemporal choice task. Among the 164 DLPFC tested in the control task, 25 neurons (15.2%) significantly modulated their activity according to the difference in the DVs for red and green targets, and this signal was significantly reduced during the control task in 11 neurons (44.0%; Figure 4C). Similarly, the majority of neurons in the striatum that showed significant modulations related to the difference in the DVs of red and green targets (ΔDV_RG_) also significantly reduced the strength of such signals during the control task (10 out of 16 neurons, or 62.5% in the CD; 8 out of 12 neurons or 66.7% in the VS; Figures 4F,I). The number of neurons showing significant interactions between ΔDV_RG_ and the task was significantly higher than expected by chance in all three areas (binomial test, *p* < 10^−7^), and all of the neurons with such significant interaction effect showed stronger signals related to ΔDV_RG_ during the intertemporal choice task.

### Time course of value signals

The results described above suggest that during the cue period of the intertemporal choice task used in the present study, neurons in the prefrontal cortex and striatum might encode the DVs in both spatial and non-spatial frames of reference in a temporally overlapping manner. We tested whether signals related to the DVs for different target colors and locations emerge with different time courses. During the task used in this study, reward magnitude was indicated by non-spatial properties, namely, colors of the targets, whereas reward delay was indicated by the number of dots surrounding the targets in the corresponding locations (Figure 1A). Therefore, DVs might be computed for red and green targets separately by first converting the signals related to reward delay in each target location to reward delay for red or green target. Alternatively, DVs might be computed for different target locations by retrieving the information about the reward magnitude from the color of the target in a given location and combining this with the signals related to reward delays in the same location.

**Figure 5:**
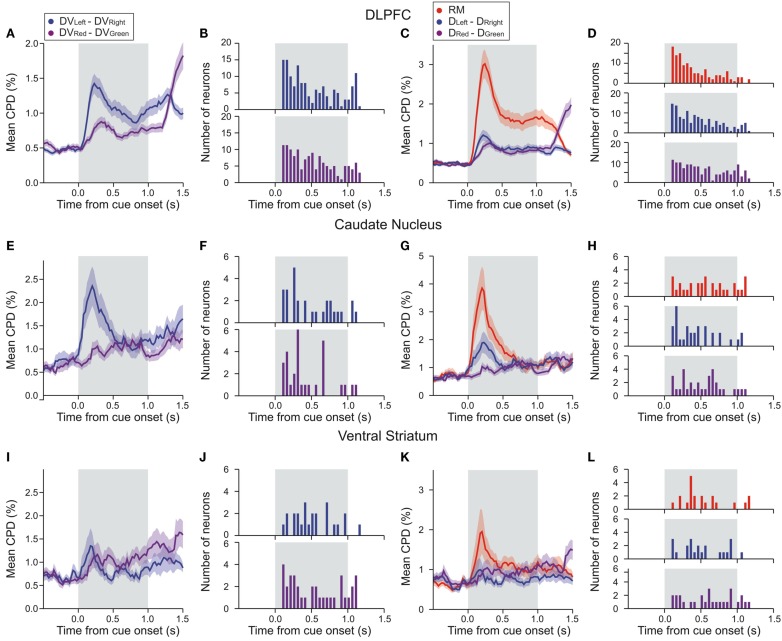
**Time course of DLPFC activity related to temporally discounted values (DVs) and reward delays in the DLPFC (A–D), CD (E–H), and VS (I–L)**. In **(A,E,I)**, average coefficient of partial determination (CPD) calculated using a 200-ms sliding window is plotted separately for the difference in the DVs for the left and right targets (blue) and the difference for the red and green targets (purple). Frequency histograms in **(B,F,G)** show the latency for the neural activity related to the difference in the DVs. In **(C,G,K)**, average CPD for the difference in the reward delays for the left and right targets (blue), the difference in the reward delays for the red and green targets (purple), and the position of the red (large reward) target (RM, red) are shown. Frequency histograms in **(D,H,L)** show the latency for the neural activity related to the difference in reward delays and the activity related to reward magnitude. Shaded areas, SEM.

**Figure 6:**
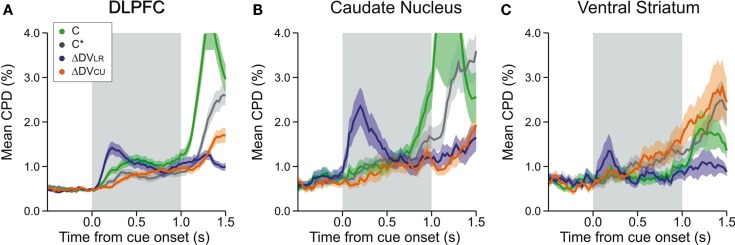
**Time course of neural activity related to different types of temporally discounted values (DVs) and the animal’s choice in DLPFC (A), CD (B), and VS (C)**. Coefficient of partial determination (CPD) was computed using a 200-ms sliding window separately for the activity related to the animal’s choice between the left and right target (*C*, green), its choice between red and green target (C*, gray), the difference in the DVs for the left and right targets (ΔDV_LR_, blue), and the difference in the DVs for chosen and unchosen targets (ΔDV_CU_, orange). Shaded areas, SEM.

In the DLPFC, signals related to the difference in the DVs of right and left target emerged significantly earlier than those related to the difference in the DVs of red and green targets (Kolmogorov–Smirnov test, *p* < 0.05; Figures 5A,B). Similarly, signals related to the difference in reward delays for two different target locations appeared in the DLPFC significantly earlier than those related to the difference in reward delays for the two different target colors (Kolmogorov–Smirnov test, *p* < 0.05; Figures 5C,D). In addition, the signals related to the relative position of red and green target tended to modulate the neural activity in the DLPFC earlier than the signals related to the reward delays (Kolmogorov–Smirnov test, *p* < 0.05; Figures 5C,D). We also found that the signals related to the difference in the DVs for left and right targets tended to appear in the DLPFC before the signals related to the position (*C*) and color (C*) of the target chosen by the animal, and also before the signals related to the difference in the DVs of the reward chosen by the animal and the other reward (Figure 6A; Kolmogorov–Smirnov test, *p* < 0.05, in all cases). These results indicate that in the DLPFC the signals necessary for selecting the action with the maximum DV (ΔDV_LR_) emerge earlier that those related to the action and its value chosen by the animal.

We applied the same analyses to the activity recorded in the striatum, but the results were less conclusive, presumably due to the smaller number of striatal neurons that showed significant effects of value-related signals (Figures 5E–L). The distribution of latencies for the differences in the DVs associated with targets presented in different colors or in different locations did not differ significantly for either CD (Figures 5E,F) or VS (Figures 5I,J). On the other hand, the signals related to the difference in the DVs of left and right targets tended to appear in the CD significantly earlier than the signals related to the position of the chosen target (Kolmogorov–Smirnov test, *p* < 0.05; Figure 6B), but this difference was not significant in the VS (Figure 6C). In addition, for the CD, the latencies of the signals related to the difference in the DVs of the red and green targets were significantly shorter than the latencies of the signals related to the position and color of the target chosen by the animal (*p* < 0.05).

### Network model

**Figure 7:**
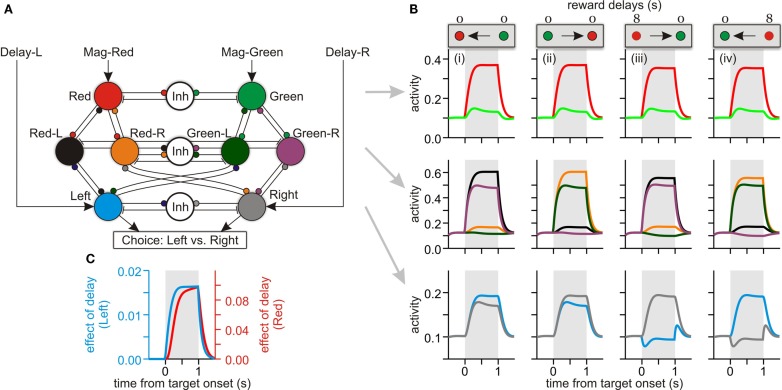
**A network model computing the temporally discounted values (DVs)**. **(A)** Pattern of connectivity within the model, with small circles and short line segments corresponding to excitatory and inhibitory connections, respectively. Units in the top layer receive the external inputs determined by the magnitude of reward expected for different target colors (red, large reward; green, small reward) and encode the DVs for red and green targets. Inputs to the middle layers (not shown) indicate the relative positions of red and green targets. For example, if the red target is presented to the right, then the inputs to units, Red-R and Green-L, are set high. Finally, the units in the bottom layer encode the DVs for left (blue) and right (gray) targets. **(B)** The activity of different units of the model during 4 sample trials in which the position of the large reward (red) target and its delay (0 or 8 s) were varied as indicated by the panels at the top (arrows indicating the target with the larger DV). Activity of different units is indicated by the same colors used in a. **(C)** Time course of activity change related to reward delay in the top (red unit) and bottom (blue/left unit) layers. This was given by the difference in the activity during the trials shown in the first and third column of **(B)**.

To account for the pattern of neural activity observed in the DLPFC during the intertemporal choice, we developed a simple network model that contains (i) a pair of units receiving signals related to the magnitude of red and green target (Figure 7A, top) and (ii) a pair of units receiving signals related to the delays of reward associated with left and right targets (Figure 7A, bottom). Therefore, the information about reward magnitude and delay was delivered to this model network as in the intertemporal choice task used in the experiment. A previous modeling study has demonstrated that value signals encoded in goods (e.g., color) space can be transformed to the value signals in spatial frame of reference via a set of units encoding the values of specific objects and locations (Soltani and Wang, 2006). Therefore, we have added similar conjunctive units in our model to propagate the signals about reward magnitude to the spatial units according to the positions of red and green targets. During the simulation, either the two units corresponding to red-left and green-right targets or those corresponding to red-right and green-left targets were activated, according to the positions of red and green targets. These conjunctive units facilitated the transmission of signals related to reward magnitude from object units to space units, and of delay signals from space units to object units. As a result, a longer delay for the reward from the red target reduced the activity of the red unit regardless of the position of the red target (Figure 7B). However, activity changes related to reward delays emerged immediately and earlier in the space units than in the object units (Figure 7C). This is not surprising, since the information about reward delays was first delivered to the space units in this model. Nevertheless, these results suggest that the time course of signals related to DVs observed in the DLPFC is consistent with the patterns expected for the network involved in converting value signals between spatial and non-spatial frames of reference.

## Discussion

### Heterogeneous neural representation of values

The subjective value or utility of reward expected from each option is influenced by multiple factors, such as the type, size, and uncertainty of reward as well as the timing of its delivery. Therefore, activity of brain areas or individual neurons involved in decision making must be influenced by the same factors that determine the utilities of different options. For example, during intertemporal choice task, the information about the magnitude and delay of reward expected from each option must be properly integrated so that the DVs from different alternatives can be compared. The results from previous single-neuron recording and neuroimaging studies suggest that this computation might be implemented in multiple brain areas. For example, single-neuron recording studies have found that the size and delay of reward or its DV modulate neural activity in multiple brain areas (Roesch et al., 2007; Kim et al., 2008; Louie and Glimcher, 2010; Cai et al., 2011). Similarly, in neuroimaging studies, signals related to values or utilities of delayed rewards are found in the VMPFC and ventral striatum (Hariri et al., 2006; Kable and Glimcher, 2007; Luhmann et al., 2008; Pine et al., 2009). However, value signals identified in neuroimaging studies were largely related to the values of the reward chosen by the subjects, although some studies demonstrated that the values associated with different types of movements can be localized to different brain areas (Wunderlich et al., 2009). Since most neuroimaging studies are likely to reflect spatial and temporal averages of underlying neural activity, they might underestimate the level of heterogeneity and true anatomical distribution of signals related to different types of utilities or value functions. In fact, when the spatial pattern, rather than the local average, of the blood-oxygen-level-dependent (BOLD) were analyzed, reward-related signals were reliably detected practically throughout the entire brain (Vickery et al., 2011). In addition, single-neuron recording studies have identified signals related to reward-related parameters in a broader range of brain regions with distinct time courses (Kable and Glimcher, 2009; Wallis and Kennerley, 2010).

Different types of value signals identified in individual neurons provide important clues about how different computational steps of decision making are implemented in the brain. For example, values of individual choices or actions have been found in multiple cortical and subcortical areas, including the prefrontal cortex, posterior parietal cortex, and basal ganglia (Platt and Glimcher, 1999; Samejima et al., 2005; Padoa-Schioppa and Assad, 2006; Kim et al., 2008; Seo et al., 2009). In addition, activity of individual neurons in these brain areas is often correlated with the sum of value signals or their difference during a binary choice task (Kim et al., 2008; Seo et al., 2009; Cai et al., 2011). Neural activity related to the difference in the value signals is likely to play a key role in decision making, since the likelihood of choosing a particular option is largely determined by the difference in the values of alternative options. It is also possible that the neural activity in some areas, such as the lateral intraparietal (LIP), might be better described by divisive normalization (Platt and Glimcher, 1999; Dorris and Glimcher, 2004; Sugrue et al., 2004; Louie et al., 2011).

Previous studies have shown that the signals related to the DVs or action values for individual targets or actions are often combined to encode their sums or differences in multiple brain areas, including the prefrontal cortex (Kim et al., 2008), posterior parietal cortex (Seo et al., 2009), and striatum (Cai et al., 2011). In the present study, we found that this might be true for the signals related to the DVs computed for different target colors or reward magnitudes. In contrast, the network model examined in the present study included only the units combining the signals related to the magnitude and delay of the reward expected from a given target location or color. Nevertheless, the units encoding the weighted averages of the value signals for multiple targets can be easily derived from the units encoding the values of individual targets.

The sum or average of values related to alternative choices reflects the overall goodness of the options, and might be used for the purpose of divisive normalization. Since this is also the expected value of reward when two alternative options are chosen equally often, signals proportional to the sum or average of values might be transformed to the value of reward expected from the animal’s chosen option during decision making (Lee et al., 2012). In fact, during intertemporal choice, neural activity in both the prefrontal cortex and striatum reflecting the sum of DVs is gradually replaced by the activity related to the DV of the reward chosen by the animal (Kim et al., 2009a; Cai et al., 2011; Kim and Lee, 2011). This suggests that the network of brain areas including these two areas is involved in translating the value signals computed for alternative options to the animal’s eventual choice. In the reinforcement learning theory (Sutton and Barto, 1998), the value of outcomes expected from a particular choice is referred to as action value functions, and they are updated on the basis of the difference between the reward expected from the animal’s chosen option and the actual reward received by the animal. Therefore, the signals related to values of chosen options, or so-called chosen values, are an important ingredient in reinforcement learning. During the intertemporal choice used in this study, there was little uncertainty about the outcomes of the animal’s choices, since the magnitude and delay of reward chosen by the animal was always clearly indicated by the visual stimuli during the cue period. Hence, it was not necessary for the animals to learn any new information during the experiment. Nevertheless, the emergence of signals related to the chosen values in the DLPFC and striatum raises the possibility that these areas might contribute to reinforcement learning when the accurate value functions must be adjusted due to the uncertainty in the animal’s environment (Barraclough et al., 2004; Lau and Glimcher, 2008; Kim et al., 2009b). Neural activity related to chosen values has been also found in other cortical areas, such as the orbitofrontal cortex, suggesting that the process of updating value functions might be broadly distributed in the brain (Padoa-Schioppa and Assad, 2006; Sul et al., 2010, 2011).

### Value signals and action selection

How value signals related to alternative options or actions in the brain are transformed to neural activity necessary for the execution of motor responses remains poorly understood, but is likely to vary depending on the nature of the behavioral task. For example, neural activity related to reward values associated with alternative actions in the prefrontal cortex, basal ganglia, and posterior parietal cortex might be a part of the process for selecting specific actions (Barraclough et al., 2004; Samejima et al., 2005; Seo et al., 2009). Although signals related to these so-called action values are found in a number of different areas, they might be first computed in a smaller number of brain areas and then broadcast to the remaining regions. This remains to be tested in future studies. In addition, values of rewards associated with different objects are often determined not only by their locations but also by their non-spatial properties. For example, properties of rewards available from different actions might be indicated by the colors of alternative targets, as in many neurophysiological studies in non-human primates (Sugrue et al., 2004; Padoa-Schioppa and Assad, 2006; Kim et al., 2008; Lau and Glimcher, 2008). In such cases, values might be first computed separately for individual objects or goods, and then subsequently transformed to the value signals associated with their locations or actions required to acquire them. During the present study, the magnitude and delay of reward available from a given target was indicated by its color and the number of dots around it. This provided an interesting opportunity to test whether the neurons in the DLPFC can represent the value signals in both spatial and non-spatial frame of reference. We found that signals related to delays for rewards available from different locations emerged significantly earlier than those related to delays for small and large rewards (corresponding to green and red targets, respectively). Accordingly, DLPFC activity tended to reflect the DVs associated with different target positions before those associated with different target colors. In addition, the signals related to the position of the chosen target appeared earlier than the color of the chosen target.

The time course of neural signals related to values of rewards associated with different objects and spatial locations might vary according to how the information about different attributes of reward is delivered during a particular behavioral task. Therefore, although we found in the present study that DLPFC neurons tended to encode the value signals in the spatial dimension earlier than those related to different objects, there are several caveats. First, in the present study, the magnitudes of large and small rewards were always fixed, although their locations were randomized across trials. As a result, it was not possible to examine the time course of neural activity related to the magnitude of reward for a given object. Second, the information about the delay of a particular reward from a given target was signaled by the number of dots presented in the same location. It is possible that DLPFC neurons might represent the delays of rewards for different objects first if the information about reward delays is integrated more tightly with other properties of the objects than their locations. More generally, the nature of value signals associated with alternative options and how this changes with the nature of task needs to be further investigated in future studies.

## Conflict of Interest Statement

The authors declare that the research was conducted in the absence of any commercial or financial relationships that could be construed as a potential conflict of interest.
